# "Anxiebo", placebo, and postoperative pain

**DOI:** 10.1186/1471-2253-5-9

**Published:** 2005-06-27

**Authors:** Paul Svedman, Martin Ingvar, Torsten Gordh

**Affiliations:** 1 Department of Plastic and Reconstructive Surgery, Malmö University Hospital, Malmö, Lund University, Sweden; 2 Department of Clinical Neuroscience, Cognitive Neurophysiology Research Group, Karolinska Institute, Stockholm, Sweden; 3 Department of Anaesthesiology and Intensive Care and Multidisciplinary Pain Center, Uppsala University Hospital, Uppsala University, Uppsala, Sweden

## Abstract

**Background:**

Surgical treatment and its consequences expose patients to stress, and here we investigated the importance of the psychological component of postoperative pain based on reports in the clinical literature.

**Discussion:**

Postoperative pain remains a significant clinical problem. Increased pain intensity with increased demand for opioid medication, and/or a relative unresponsiveness to pain treatment was reported both when the analgesia was administered by means of conventional nurse injection regimes and patient-controlled analgesia (PCA). Both the quality of the analgesia, and the sensitivity of postoperative models for assessing analgesic efficacy could be significantly influenced.

The findings could be explained by increased penetration of an algesic anxiety-related nocebo influence (which we chose to call "anxiebo") relative to its analgesic placebo counterpart. To counteract this influence, the importance of psychological effects must be acknowledged, and doctors and attending nurses should focus on maintaining trustful therapist-patient relationships throughout the treatment period. The physical mechanism of anxiebo should be further explored, and those at risk for anxiebo better characterized. In addition, future systemic analgesic therapies should be directed towards being prophylactic and continuous to eliminate surgical pain as it appears in order to prevent the anxiebo effect.

Addressing anxiebo is the key to developing reproducible models for measuring pain in the postoperative setting, and to improving the accuracy of measurements of the minimum effective analgesic concentration.

**Summary:**

Anxiebo and placebo act as counterparts postoperatively. The anxiebo state may impair clinical analgesia and reduce the sensitivity of analgesic trials. Ways to minimize anxiebo are discussed.

## Background

In spite of considerable progress in postoperative analgesia, recent studies show that adequate pain relief remains elusive for a significant fraction of hospitalized surgical patients [1-3]. Continuous awareness of this problem and further efforts to improve treatment are required despite the availability of acute pain services [3]. Surgical treatment and its consequences cause psychological stress, and pain intensity after surgery is influenced by both analgesic drug effects and psychological factors. These factors comprise on the one hand an analgesic placebo influence induced by the therapeutic situation *per se*, and on the other hand its clinically less studied algesic anxiety-related nocebo influence counterpart [4-6] (in this article the term "anxiebo" is used to better focus on how to deal with this connection clinically).

The clinical effects of anxiebo have been only incompletely evaluated. In this work, relevant reports in the literature were assessed with regard to evidence of anxiebo, and its clinical expressions are described here. Implications of the findings on clinical practice, development of analgesic drugs, and assay sensitivity of clinical trials are discussed.

## Discussion

### The anxiebo-placebo relationship

**Figure 1:**
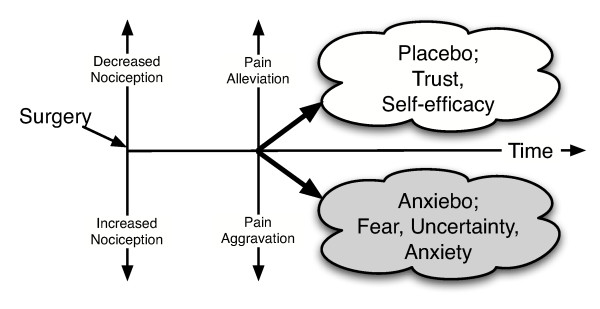
Factors influencing postoperative anxiebo or placebo.

The levels of perceived self-control (self-efficacy) and anticipatory anxiety are important factors in determining whether somebody will be a placebo or anxiebo responder [7-9], and may be influenced by the presence or absence of supportive social interaction. In this respect, the central role of the therapist/patient relationship should be recognized. Anxiebo may result from either a lack of supportive social interaction or inherently weak self-control in connection with exposure to painful stimuli (Figure 1). The lower the overall experience of control, the stronger the reported anticipatory anxiety, the pain experience expressed as pain-intensity ratings, and the autonomic activation.

The effects of anxiebo and placebo can be clearly visualised on PET scans of the brain [9]. During placebo analgesia the activity patterns in the brain, brainstem and descending antinociceptive systems demonstrate that the endogenous opioid system is activated, and there are indications that the placebo state is sensitive to the effects of morphine [9,10]. These findings suggest that a placebo patient under opioid treatment should be able to closely approach a pain-free state. In anxiebo, on the other hand, scan findings indicate that the sensitivity to opioids is reduced but not abolished [9,11].

Reviews of analgesic trial outcomes have indicated that the degree of pain relief obtained may range between 0 and 100 per cent both for placebo and active drug patients [12,13]. A plausible explanation for this is that placebo and anxiebo act reciprocally and that the degree of shift in either direction is determined by how psychological elements of the analgesia are handled.

### Evidence of anxiebo in postoperative analgesia

A combination of nonopioid analgesics and opioids is most often used to achieve postoperative analgesia, and the opioids are frequently administered by nurse-administered injections. Delay between need and injection constitutes the major problem with this approach [2]. Classic references indicate that preoperative encouragement and preparation of patients receiving nurse injections may decrease their opioid dosage by 50% [14,15], and directed patient information and psychological support are advocated. There is a moderate correlation between variable degrees of anxiety on the one hand, and pain intensity and requirement for opioid analgesics on the other, and these effects appear not only when the opioid injections are administered by an attendant nurse but also in patient-controlled analgesia (PCA) [16], where the access to intermittent doses of opioid should not be a limitation. While morphine at low doses is used as an anxiolytic, a deeper analysis of this effect, for instance with regard to the dose-response relationship and individual variation, appears to be lacking. Other approaches to reducing anxiety include the preoperative use of benzodiazepine [17] or distracting the patient, for instance by exposure to music [18]. Furthermore, a study of dental out-patients has suggested that a postoperative telephone call from the therapist demonstrating care and reassurance may improve the analgesia [19].

Several studies indicate that a significant fraction of PCA patients experiences moderate or severe pain [1,3]. Regional nerve blocks – which also require more doctor-patient interaction – on average produce clearly better results than systemic opioid techniques [2]. Even considering pain components that are known to be opioid resistant, the only way of explaining why timely treatment with systemic opioids should leave a proportion of patients with these levels of residual pain is by implicating a degree of anxiebo. This link is further confirmed by a controlled study that showed that PCA in the early phase after knee surgery (performed under general anesthesia) was associated with low oxygen tension in the subcutis, while a normal tension was observed after more-effective regional analgesia [20]. These findings indicate different levels of psychological stress and sympathetic tone in the two patient groups, and the oxygen tension in the group that received only the less-effective opioid analgesia was so low that it predisposed to surgical wound infection.

Studies into the use of PCA lend themselves also to further evaluation because of the considerable use of PCA as a research technique for assessing analgesic efficacy. There are many indications in the literature that PCA functions well as an analgesic method and that the patient-control concept is accepted by the patients. However, the question of control may be difficult to assess in a clinical situation involving an element of dependence. Recent reports show that many PCA patients do not recognize themselves as being in control of their treatment, and they may experience side effects and fear on utilization that limit their ability to control pain [21,22]. It is well known also from other studies that several psychological parameters are predictors of pain and/or opioid use (i.e. the propensity to press the controlling button) in PCA [16], of which anxiety-related factors are common. Thus, the connection between anxiebo and opioid use may explain why 20% of patients using PCA were found to press the analgesic-delivery button at an unchanged rate when the injected opioid dose was decreased [23]. Against this background, the findings in 18 trials assessing the efficacy of continuous basal or therapeutic infusions of opioid in connection with PCA are of interest. In the two largest studies [24,25], the addition of infusions at different rates did not reduce pain intensity, while the injected PCA dosages were clearly reduced in one study but not in the other. The remaining (smaller) studies demonstrated neither clinically convincing reductions in PCA dosage nor reductions in pain intensity that might be of general importance. Assuming full sensitivity to the continuously administered opioid, one should have expected the pain intensity to approach zero with increasing dosage, and as a result reproducible decreases in PCA analgesic dosage. The findings indicate that the assumption that the patients titrate to a common pain intensity level, and adapt their injection rates to an added analgesic stimulus, may have important limitations. It is therefore reasonable to implicate anxiebo in these examples of remarkable and long-lasting unresponsiveness to a continuous dosage of opioid. The underlying causes of these indications of poor sensitivity to the effect of opioids have, to our knowledge, not been further explored in the clinical literature.

In PCA, it is logical that the combination of an anxiebo-induced decrease in opioid sensitivity and an increase in the rate of self-administration as part of a coping reaction to anxiebo may in certain patients lead to pronounced overuse of morphine that is unrelated to the need for analgesia.

### Implications for therapy, drug development, and research

**Figure 2:**
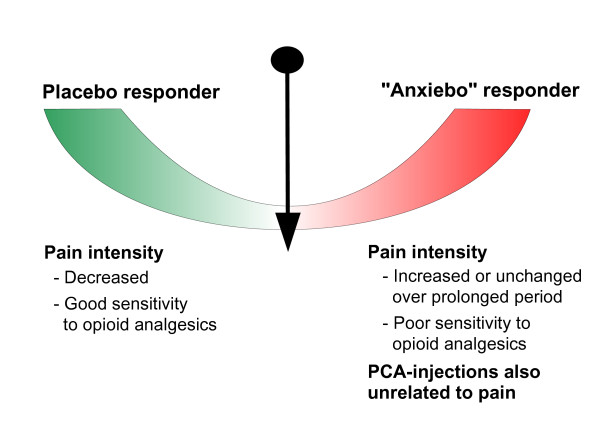
**Reciprocal effects of anxiebo and placebo in postoperative patients. **The propensity for a fraction of patients with patient-controlled analgesia (PCA) to press the analgesic-delivery button for reasons unrelated to pain may reflect a coping mechanism.

The observations above indicate that both nurse injection regimes and PCA may produce signs of anxiebo characterized by increased pain intensity with increased demand for opioid medication, and/or a relative unresponsiveness to pain treatment, which may last for days (Figure 2). Avoiding anxiebo and thereby promoting placebo thus appears to be an important component of effective analgesic treatment. In order to accomplish this, the central role of a trustful therapist-patient relationship (doctor and nurse *vs *patient) throughout the treatment period should be recognized. Assuming that anxiebo could be eliminated clinically, the placebo state would be more prevalent, although its maximal overall influence in terms of improving the outcome of opioid treatment cannot yet be judged.

It is likely that the direct caregiver-patient relationship in nurse injection regimes can be used to better advantage, and minimizing stressful break-through pain should be emphasized. With regard to PCA, we do not doubt its role when applied in a suitable, psychologically supportive environment, but the method is more complex than generally thought. For those patients who do not adapt to the control concept, and respond with signs of anxiebo, extra support may be required or alternative treatment should be sought.

Conceptually, it would appear advantageous to direct future systemic analgesic therapies towards being prophylactic and continuous (partly to eliminate surgical pain as it appears, and partly to prevent the anxiebo effect) rather than reactive and intermittent. By analogy, the use of continuously acting psychotropic drugs that specifically counteract elements of the anxiety state may be effective in patients who respond poorly to analgesia.

The mechanisms underlying anxiebo should be further explored and other measures aimed at reducing anxiebo should be examined. In particular, further efforts should be devoted to defining patient groups and individuals that are especially at risk. This may be done by developing easily applicable scales for identifying at-risk patients preoperatively, and by preparing these patients with suitable information. Techniques should be sought which allow postoperative diagnosis of anxiebo. We suggest that perceived expectancy of future pain as opposed to subjective estimates of current pain may be a parameter of interest for better predicting a patient's risk for the anxiebo effect.

While the drug placebo effect is regularly accounted for in clinical trials, the effect of its anxiebo counterpart is not. It appears likely that trial assay sensitivity is reduced with variable penetration depending on the specific therapeutic circumstances of the individual study. Anxiebo may result in false-negative or borderline results, as indicated above for PCA models. Such systemic errors are not removed by simply increasing the size of the trial. The key to a reproducible, sensitive model is the proper handling of individual factors interacting within the psychological context, with respect for the therapist-patient relationship. Education and support to personnel handling the patients postoperatively seems to be the critical factor. Optimally, the anxiebo effect should be accounted for, perhaps by conducting a preliminary sensitivity test using a drug (such as morphine) with known analgesic effects.

Since patients or patient groups receiving PCA may not titrate their opioid dosage (Figure 2), it follows that the general usability of the clinical concept of minimum effective analgesic concentration as a guide for dosage can be questioned in particular studies.

## Summary

The importance of psychological reactions are commonly acknowledged in postoperative analgesia, but the way these reactions express themselves and the degree of disturbance they may cause by producing anxiebo rather than placebo states are at present incompletely considered. Maintaining trustful therapist-patient relationships throughout the treatment period is very important. Future systemic analgesic therapies should be directed towards being prophylactic and continuous to eliminate surgical pain as it appears so as to prevent the anxiebo effect. The physical mechanism of anxiebo should be further explored, and more effort made to define patient groups and individual patients especially at risk. Addressing anxiebo is of importance also in the development of reproducible models for assessing analgesic efficacy in the postoperative setting, and may improve the accuracy of measurements of the minimally effective analgesic concentration.

## Competing interests

The author(s) declare that they have no competing interests.

## Authors' contributions

All three authors participated in the design and preparation of the manuscript, and all approved the final draft.

## Pre-publication history

The pre-publication history for this paper can be accessed here:


